# Supplementary material for: Gate-set evaluation metrics for closed-loop optimal control on nitrogen-vacancy center ensembles in diamond
Source: npj Quantum Inf. 2024 Oct 2;10(1):96. doi: 10.1038/s41534-024-00893-y (PMC11446828; doi:10.1038/s41534-024-00893-y)
Supplement: Supplementary file 1 — Supplementary Information [file 41534_2024_893_MOESM1_ESM.pdf]

Supplementary Information: Gate-set evaluation metrics for closed-loop optimal control on nitrogen-vacancy center ensembles in diamond

Philipp J. Vetter,[1,2,*] Thomas Reisser,[3,4,*] Maximilian G. Hirsch,[1,2,5] Tommaso Calarco,[3,4,6] Felix Motzoi,[3,4] Fedor Jelezko,[1,2] and Matthias M. Müller[3]

[1] *Institute for Quantum Optics, Ulm University, Albert-Einstein-Allee 11, 89081 Ulm, Germany*
[2] *Center for Integrated Quantum Science and Technology (IQST), 89081 Ulm, Germany*
[3] *Peter Grünberg Institute – Quantum Control (PGI-8), Forschungszentrum Jülich GmbH, D-52425 Germany*
[4] *Institute for Theoretical Physics, University of Cologne, D-50937 Germany*
[5] *Current address: NVision Imaging Technologies GmbH, Wolfgang-Paul-Straße 2, 89081 Ulm, Germany*
[6] *Dipartimento di Fisica e Astronomia, Università di Bologna, 40127 Bologna, Italy*

(Dated: March 2024)

CONTENTS

I. Hilbert-Schmidt space 1

II. Fluence 2

III. Maximum-Likelihood Estimation 2

IV. Randomized benchmarking experiments 3

V. Optimization Metrics 4

VI. FoM progression during the optimizations 5

VII. Gain under Amplitude Changes 6

References 6

I. HILBERT-SCHMIDT SPACE

To simplify the notation throughout our manuscript we express our states and operations in the so-called *Hilbert-Schmidt* (HS) space [1, 2].
For this, we normalize the Pauli matrices by their dimension $P_i \to P_i/\sqrt{d}$ where $d = 2$ and choose them as our basis

$$P_k \in \left\{ \frac{\mathbb{1}}{\sqrt{2}}, \frac{\sigma_x}{\sqrt{2}}, \frac{\sigma_y}{\sqrt{2}}, \frac{\sigma_z}{\sqrt{2}} \right\}. \tag{1}$$

Density matrices are then written as d^2 vectors identified by double bra or ket brackets

$$|\rho\rangle\rangle = \sum_k |P_k\rangle\rangle\langle\langle P_k|\rho\rangle\rangle = \sum_k |P_k\rangle\rangle \ \mathrm{tr}\{P_k^\dagger \rho\}, \tag{2}$$

whereas the Hilbert-Schmidt inner product is given by

$$\langle\langle \alpha|\beta\rangle\rangle = \mathrm{tr}\{\alpha^\dagger \beta\} \ . \tag{3}$$

Operators describing linear maps can be written in the HS space as $d^2 \times d^2$ operators

$$\mathcal{O}_\Lambda = \sum_{jk} |P_j\rangle\rangle\langle\langle P_j|\hat{\mathcal{O}}_\Lambda|P_k\rangle\rangle\langle\langle P_k| \tag{4}$$

* These authors contributed equally to this work.
Corresponding author: philipp.vetter(at)uni-ulm.de

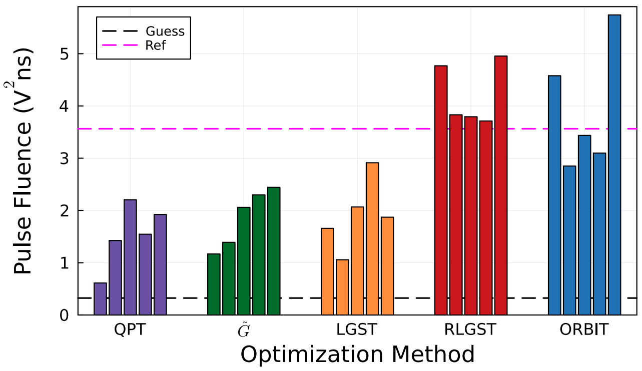

FIG. 1. Fluence of the optimized pulses. We perform five optimization runs per method shown in the x-axis. The fluence of the final optimized pulse is shown by the height of the individual bars. The black dashed line corresponds to the fluence of the guess pulse and the magenta one to the fluence of the reference pulse.

with $\langle\langle P_j|\hat{\mathcal{O}}_\Lambda|P_k\rangle\rangle = \mathrm{tr}\{P_j\Lambda(P_k)\}$. In our case, the linear map is simply given by the corresponding unitary pulse gate, $\Lambda(P_k) = UP_kU^\dagger$.
A measurement of a POVM $\langle\langle E|$ of the action of the gate G on the initial state $|\rho\rangle\rangle$ is then given by

$$p_G = \langle\langle E|G|\rho\rangle\rangle. \tag{5}$$

II. FLUENCE

To better understand why certain methods achieve significantly higher and consistent gains than others, we calculate the fluence of our optimized pulses. The fluence is given by

$$\Gamma = \int_0^{t_p} \left(a_x^2(t) + a_y^2(t)\right) \mathrm{d}t, \tag{6}$$

with the time-dependent amplitudes $a_x^2(t)$ and $a_y^2(t)$ of Eq. (3) from the main text and the pulse length t_p. While we ensure that at no point any pulse can have an amplitude larger then the reference pulse, the optimized pulse can achieve a higher fluence due to its longer pulse length.
The results for each optimization are shown in Fig. 1, where the height of the bars denote the fluence of the corresponding optimized pulse. For RLGST and ORBIT we observe on average a slightly higher fluence than for the reference pulse, while the fluence of all other methods is located between the one of the reference and the guess pulse. To achieve their enhanced robustness for any investigated method, RLGST and ORBIT thus require a significantly higher fluence than the other methods. The increased fluence seems to be a perquisite for the gate-set's successful application at long time-scales, which is in line with the observations in the main text. The connection between fluence and gate-performance stems from the higher amplitudes allowed for the corresponding pulses. While a low fluence restricts the pulse, a higher amplitude allows for a enhanced robustness with respect to frequency errors as they are suppressed in the effective Rabi frequency $\sqrt{\Omega^2+\Delta^2}$, with Ω being the applied Rabi frequency (= microwave amplitude) and Δ the detuning.
The optimized pulses of $\tilde{G}$ show a much smaller fluence compared to the reference pulse. Yet, the method shows a significantly higher gain than the reference pulse when evaluated by itself. This makes the method particularly suitable if heating imposes an experimental limitation and the gate-set is to be optimized for specific applications of the gates to arbitrary basis states.

III. MAXIMUM-LIKELIHOOD ESTIMATION

Typically, the estimates provided by LGST are used as starting points for a maximum-likelihood estimation (MLE) to obtain real, physical estimates of the applied gates and states. For such an MLE we take the expectation values p_{ijk} (see Eq. (7) in the main text) measured with LGST and minimize

$$\sum_{ijk}\left(p_{ijk} - \sum_{mnrstu} \left(\chi_{F_i}\right)_{tu} \left(\chi_{G_k}\right)_{rs} \left(\chi_{F_j}\right)_{mn} \mathrm{tr}\{EP_tP_rP_m\rho P_nP_sP_u\}\right) \tag{7}$$

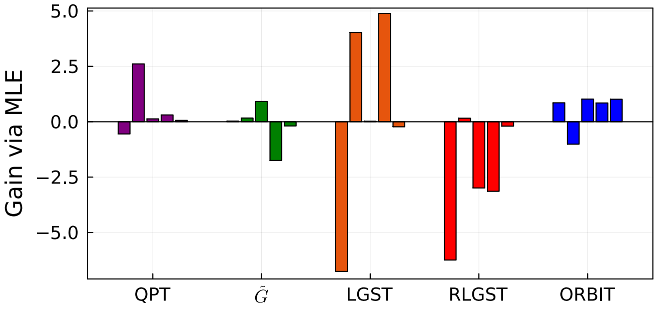

FIG. 2. Evaluation of the different optimization runs through MLE. The abscissa shows the chosen optimization method, the ordinate the achieved gain.

constrained by

$$\begin{aligned} &\sum_{mn} (\chi_G)_{mn} \operatorname{tr}(P_m P_r P_n) - \delta_{0r} = 0\,,\ \forall\, G \in \mathcal{G} \\ &\operatorname{tr}\{\rho\} = 1\,, \\ &\mathbb{1} - E \succcurlyeq 0 \end{aligned} \tag{8}$$

according to Ref. [2]. The process matrix χ_G describes the action of a gate on a given density matrix by

$$\Lambda(\rho) = \sum_{i,j=1}^{d^2} (\chi_G)_{ij}\ P_i \rho P_j \tag{9}$$

with the Pauli operators P_i, P_j. Performing the MLE for 20 repeated measurements of a fixed set of pulses results in a variance of about 1% for the obtained process matrix fidelities. From the LGST estimates of the gates one can calculate estimates for the process matrices, which are then used as a starting point for the minimization. We perform the MLE for the five optimization runs per method. The process matrices resulting from the MLE are compared to the target process matrices to determine their fidelity. Using the sum of the individual fidelities to calculate the optimization gain according to Eq. (2) in the main text leads to the results shown in Fig. 2. We observe a huge variance between different optimization runs for one method, as well as extremely large and small gain values. If we use the target process matrices as a starting point for the minimization such that the LGST estimates do not negatively influence the minimization, we obtain a similar result. This is in stark contrast to all other evaluation methods and is reminiscent of the results from the evaluation with LGST. The measurement errors of our experiment seem to strongly influence the MLE and do not allow us to to clearly distinguish between the gate-set of the guess, reference and optimized pulse. For this reason, the MLE is omitted as evaluation method.

IV. RANDOMIZED BENCHMARKING EXPERIMENTS

We additionally use randomized benchmarking (RB) [3–5] to evaluate our optimized gate-sets performance. An exemplary measurement is shown in Fig. 3, where the gate-set was optimized using ORBIT. Heating limits us to a maximum circuit length of 18 Cliffords and we average over 300 randomized circuits for each circuit length. The survival probability $p_s(m)$ is fitted with

$$p_s(m) = A \cdot q^m + B, \tag{10}$$

where m corresponds to the number of Cliffords. Due to our normalization, all SPAM errors are absorbed by A, leading to $B = 0.5$ by definition. The parameter q is used to calculate the average error per Clifford according to Ref. [4, 5]. For the shown example we obtain an average error rate per Clifford of $r = 0.0258 \pm 0.0007$ with $A = 0.430 \pm 0.008$.

Tab. I shows the average error rates per gate for the optimized gate-sets, i.e. the average error rate per Clifford, as extracted from RB, converted to native gates.

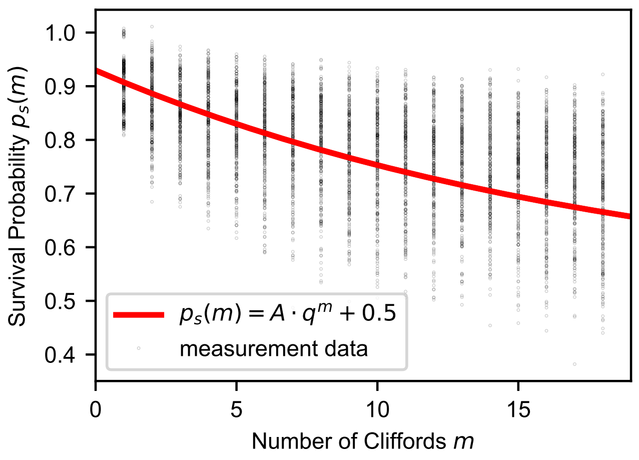

FIG. 3. Randomized benchmarking experiment for an optimized gate-set. The abscissa shows the number of applied Clifford gates m and the ordinate shows the corresponding survival probability $p_s(m)$. The data is fitted using a single exponential decay illustrated by the red line.

TABLE I. Average error rate per native gate for the optimized pulses.

opt. method	run 1	run 2	run 3	run 4	run 5
QPT	0.0142 ± 0.0005	0.0105 ± 0.0004	0.0089 ± 0.0004	0.0108 ± 0.0004	0.0100 ± 0.0005
$\tilde{G}$	0.0094 ± 0.0004	0.0105 ± 0.0005	0.0093 ± 0.0004	0.0094 ± 0.0004	0.0092 ± 0.0004
LGST	0.0131 ± 0.0005	0.0163 ± 0.0006	0.0133 ± 0.0005	0.0130 ± 0.0005	0.0127 ± 0.0005
RLGST	0.0088 ± 0.0004	0.0088 ± 0.0004	0.0088 ± 0.0004	0.0118 ± 0.0005	0.0096 ± 0.0004
ORBIT	0.0081 ± 0.0004	0.0091 ± 0.0004	0.0082 ± 0.0004	0.0093 ± 0.0004	0.0095 ± 0.0004

V. OPTIMIZATION METRICS

Tab. II shows the average number of evaluation steps of our optimizations for the selected method. RLGST and ORBIT require notably more evaluation steps than an optimization with QPT, $\tilde{G}$ or LGST. The parameter landscape of those two methods must thus be significantly more complex, requiring more evaluation steps until the optimizer converges according to the set stopping criteria.

In addition, the average duration of one evaluation step for ORBIT and RLGST is also significantly longer than for the other methods. To obtain the corresponding FoM of the two methods, we average over 300 circuits, i.e. we perform a measurement sequence which contains 236 more measurements than e.g. for $\tilde{G}$. These additional measurements increase the overall length of the measurement sequence, leading to a longer uploading time to the AWG and thus to a longer mean evaluation step duration.

TABLE II. Optimization Metrics. The average number of evaluation steps, their average duration and the average total optimization time per method is displayed together with the corresponding uncertainty.

method	mean number of evaluation steps	mean evaluation step duration (s)	mean optimization duration (h)
QPT	872 ± 26	40.524 ± 0.028	10.3 ± 0.4
$\tilde{G}$	880 ± 80	46.74 ± 0.17	14.1 ± 1.8
LGST	723 ± 9	47.25 ± 0.16	11.5 ± 0.8
RLGST	1220 ± 60	66.49 ± 0.25	32 ± 5
ORBIT	1100 ± 100	67.3 ± 0.4	28.0 ± 2.9

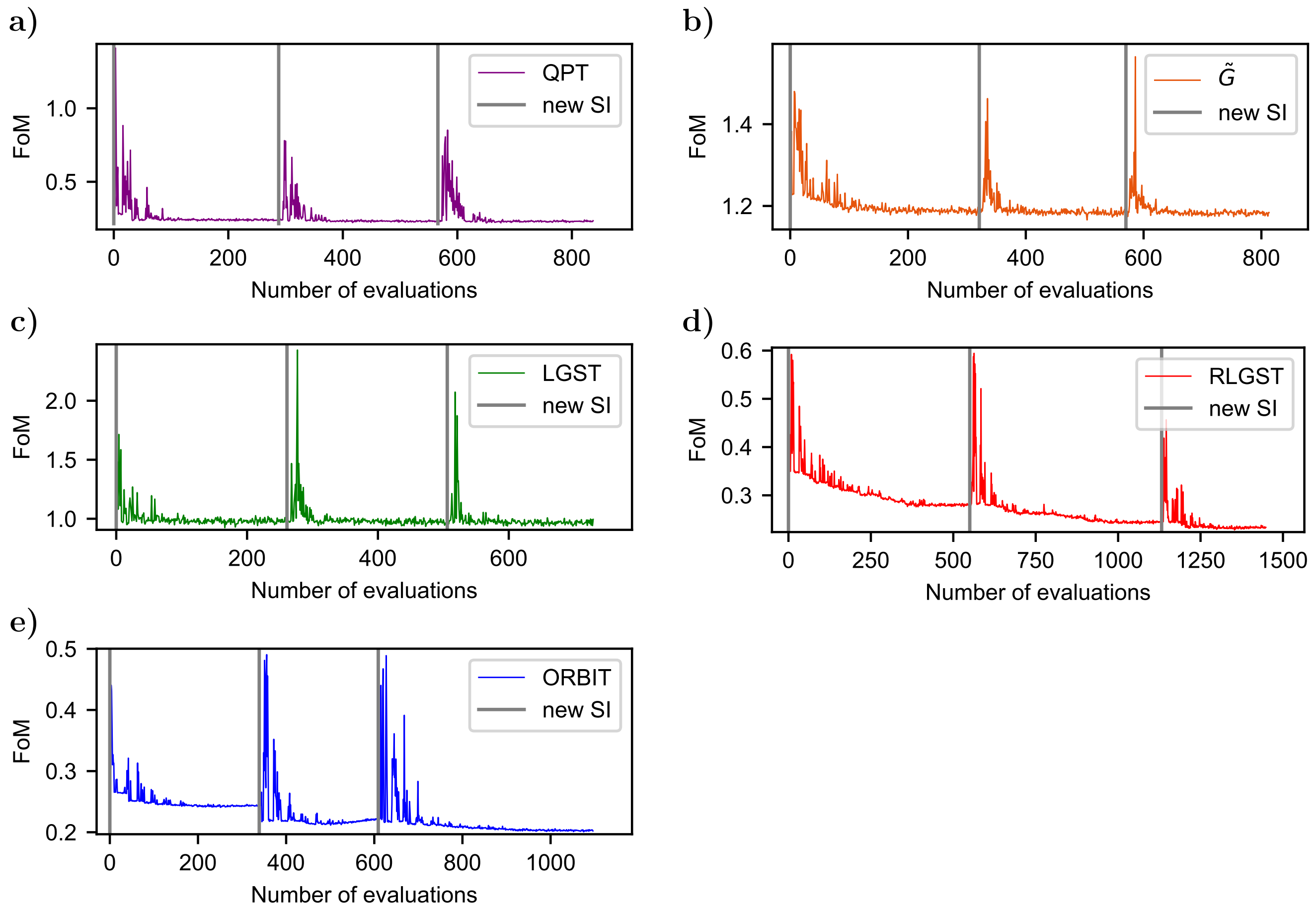

FIG. 4. FoM progression. Exemplary FoM progression during an optimization via **a)** QPT, **b)** $\tilde{G}$, **c)** LGST, **d)** RLGST and **e)** ORBIT. The abscissa shows the number of evaluations and the ordinate the absolute value of the corresponding FoM. The grey lines mark the beginning of a new super-iteration.

VI. FOM PROGRESSION DURING THE OPTIMIZATIONS

Fig. 4 shows the FoM progression during an optimization for the different analysis methods. The optimization via QPT in Fig. 4 a) shows a clear minimization of the FoM. We observe a strong modulation of the FoM during the beginning of a new super-iteration. In addition, the FoM converges very quickly and shows almost no variance between different super-iterations.

For the optimization via $\tilde{G}$ in Fig. 4 b) we also see a clear minimization but the ratio between the final FoM and the one of the initial guess is small compared to the other methods. Depending on the optimization run we can observe a further decrease of the FoM through additional super-iterations.

For LGST in Fig. 4 c) we observe again strong variations of the FoM at the beginning of a super-iteration. The improvement of the FoM is almost within the noise, due to the problems discussed in the main text. QuOCS is well equipped for such a task through the use of re-evaluation steps to correctly determine if the FoM truly improved or not. This allows us to enhance the gate-set's performance even for LGST.

For RLGST in Fig. 4 d) and ORBIT in Fig. 4 e) we observe a very clear minimization of the FoM from the initial guess. The FoM seems to be well defined such that QuOCS can easily improve the pulse shape and converge within the set boundaries. One can see a clear improvement of the FoM through the use of additional super-iterations. As we limit ourselves to three super-iterations due to time-limitations, we cannot exclude that the gains reported in the main text could be much higher for longer optimizations.

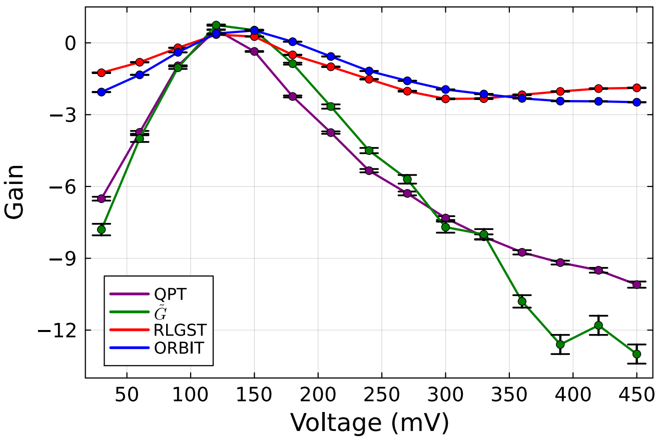

FIG. 5. Measured gain values for the different methods under a sweep of the applied voltage. Each data point shows the mean value of 20 measurements with the corresponding standard deviation.

VII. GAIN UNDER AMPLITUDE CHANGES

Fig. 5 shows the gain of the different evaluation methods for a sweep of the driving field voltage for the rectangular guess pulse. An increase or decrease from the ideal voltages corresponds to an over- or under-rotation of the gate and leads to a fall-off of the gain for each method.

As over- and under-rotations are classified as coherent errors, the steeper descent of QPT and $\tilde{G}$ indicates that these methods are more sensitive to those types of errors than RLGST and ORBIT. Due to the different weighting of these errors in the respective FoM, the curves also show different positions of the maxima.

[1] A. Y. Kitaev, A. H. Shen, and M. N. Vyalyi, Classical and Quantum Computation (American Mathematical Society, USA, 2002).

[2] D. Greenbaum, Introduction to quantum gate set tomography, arXiv preprint arXiv:1509.02921 (2015).

[3] E. Knill et al., Randomized benchmarking of quantum gates, Phys. Rev. A **77**, 012307 (2008).

[4] E. Magesan, J. M. Gambetta, and J. Emerson, Scalable and robust randomized benchmarking of quantum processes, Phys. Rev. Lett. **106**, 180504 (2011).

[5] E. Magesan, J. M. Gambetta, and J. Emerson, Characterizing quantum gates via randomized benchmarking, Phys. Rev. A **85**, 042311 (2012).
